# The acute and long-term management of anaphylaxis: protocol for a systematic review

**DOI:** 10.1186/2045-7022-3-14

**Published:** 2013-04-10

**Authors:** Sangeeta Dhami, Sukhmeet S Panesar, Tamara Rader, Antonella Muraro, Graham Roberts, Margitta Worm, Aziz Sheikh

**Affiliations:** 1 Evidence-Based Health Care Ltd, 113 The Murrays, Edinburgh EH17 8UD, UK; 2 Medical School, Doorway 3, University of Edinburgh, Teviot Place, Edinburgh EH8 9AG, UK; 3 University of Ottawa, 75 Laurier Avenue East, Ottawa, ON K1N 6N5, Canada; 4 Padua General University Hospital, Via Giustiniani 3, Padua 35128, Italy; 5 Faculty of Medicine, University of Southampton, Southampton SO171BJ, UK; 6 Charité University, Charitestraße 1, Berlin 10117, Germany

**Keywords:** Anaphylaxis, Management, Allergy, Emergency

## Abstract

**Background:**

The European Academy of Allergy and Clinical Immunology is in the process of developing its Guideline for Food Allergy and Anaphylaxis, and this systematic review is one of seven inter-linked evidence syntheses that are being undertaken in order to provide a state-of-the-art synopsis of the current evidence base in relation to epidemiology, prevention, diagnosis and clinical management and impact on quality of life, which will be used to inform clinical recommendations.

The aims of this systematic review will be to assess the effectiveness of interventions for the acute management of anaphylaxis, and pharmacological and non-pharmacological approaches for the long-term management of anaphylaxis.

**Methods:**

A highly sensitive search strategy has been developed, and validated study design filters will be applied to retrieve all articles pertaining to the management of anaphylaxis from electronic bibliographic databases. We will systematically review the literature on the acute management of anaphylaxis by assessing the effectiveness of epinephrine, H1-antihistamines (versus placebo), systemic glucocorticosteroids, methylxanthines or any other treatments for the emergency management of people experiencing anaphylaxis. The main interventions that have been studied in the context of long-term management are anaphylaxis management plans and allergen-specific immunotherapy.

**Discussion:**

There is at present little in the way of robust evidence to guide decisions on the acute and/or long-term management of anaphylaxis. Given the risk of death and the considerable morbidity associated with anaphylaxis these evidence gaps need to be filled wherever possible; this systematic review will make a start in this area.

## Background

Anaphylaxis can be defined as a “severe, life-threatening generalised or systemic hypersensitivity reaction” [1,2]. Several working definitions of anaphylaxis have been formulated to aid clinical diagnosis and management [3-6]. The most well-known of these is the consensus clinical definition proposed by Sampson et al., which involved representatives of a number of international allergy organisations, including the European Academy of Allergy and Clinical Immunology (EAACI) [7].

Management considerations centre on the acute, emergency treatment of reactions and longer-term management considerations, which aim to reduce the risk of further reactions and improve outcomes if, despite these measures, a further reaction ensues [8]. Although a self-limiting condition in the majority of cases, it is at present difficult to predict the severity of future reactions. Fatalities often occur within minutes of the onset of a reaction; underscoring the importance of effective, emergency management of reactions [9,10]. The prompt administration of epinephrine (adrenaline) is particularly important, but although potentially life-saving, evidence indicates that this is under-used both by patients/carers and professionals [11]. A range of second-line treatments are also sometimes recommended [12]. There are furthermore a number of pharmacological and non-pharmacological approaches that can be used to try and minimise future risk, but the effectiveness and safety of these management strategies is in many cases unclear [13].

The EAACI is in the process of developing the EAACI Guideline for Food Allergy and Anaphylaxis, and this systematic review is one of seven inter-linked evidence syntheses that are being undertaken in order to provide a state-of-the-art synopsis of the current evidence base in relation to epidemiology, prevention, diagnosis and clinical management and impact on quality of life, which will be used to inform clinical recommendations.

### Aims

The aims of this systematic review will be to assess the effectiveness of interventions for the:

• Acute management of anaphylaxis

• Pharmacological and non-pharmacological approaches for the long-term management of anaphylaxis.

## Methods

### Search strategy

**Figure 1:**
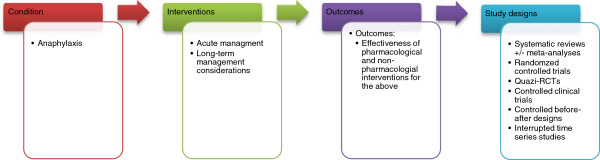
Conceptualisation of systematic review of interventions for the acute and longer-term management of anaphylaxis.

A highly sensitive search strategy has been developed, and validated study design filters will be applied to retrieve all articles pertaining to the management of anaphylaxis from electronic bibliographic databases. We have conceptualised the search to incorporate three elements, as shown in Figure 1.

To retrieve systematic reviews, we will use the systematic review filter developed at McMaster University Health Information Research Unit (HIRU) [14]. To retrieve randomised controlled trials (RCTs), we will apply the Cochrane highly sensitive search strategy for identifying randomised trials in MEDLINE: sensitivity- and precision-maximising version (2008 revision); Ovid format from Chapter 6 of the Cochrane Handbook [15]. To retrieve non-randomised studies, i.e. controlled clinical trials (CCT), controlled before-and-after (CBA) and interrupted time-series (ITS) studies, we will use the Cochrane Effective Practice and Organisation of Care (EPOC) filter Version 2.4, available on request from the EPOC Group [16,17]. To retrieve case series, we used the filter developed by librarians at Clinical Evidence [18].

We will search the following databases:

• Cochrane Library including,

◦ Cochrane Database of Systematic Reviews (CDSR)

◦ Database of Reviews of Effectiveness (DARE)

◦ CENTRAL (Trials)

◦ Methods Studies

◦ Health Technology Assessments (HTA)

◦ Economic Evaluations Database (EED)

• MEDLINE (OVID)

• Embase (OVID)

• CINAHL (Ebscohost)

• ISI Web of Science (Thomson Web of Knowledge)

• TRIP Database (http://www.tripdatabase.com)

• Clinicaltrials.gov (National Institutes of Health web).

The search strategy has been developed on OVID MEDLINE and then adapted for the other databases (see Additional file 1 for full search strategies). In all cases the databases will be searched from inception to 30 September 2012. All references will be imported into an EndNote Library and tagged with the name of the database. Additional references will be located through searching the references cited by the identified studies, and unpublished work and research in progress will be identified through discussion with experts in the field. We will invite experts who are active in the field from a range of disciplines and geography to comment on our search strategy, and the list of included studies. There are no language restrictions and, where possible, all literature will be translated.

### Inclusion criteria

• Systematic reviews +/− meta-analyses

• RCTs

• Quasi-RCTs

• CCTs

• CBA designs

• ITS studies

• Case-series (for studies investigating the use of epinephrine, and with a minimum of 10 patients)

We will appraise the evidence by looking at higher levels of evidence such as systematic reviews and/or meta-analyses of RCTs, together with individual RCTs. However, we suspect that there will be limited information available so we have opted to include quasi- RCTs and CCTs (where non-random allocation of patients has occurred), and EPOC study designs such as CBA study designs (observations are made before and after the implementation of an intervention) and ITS studies (where observations at multiple time-points before and after and intervention are made) [19]. Case-series, despite being lower forms of evidence, will be included as advice from experts at the EAACI suggests that studies looking at the use of epinephrine for managing acute anaphylaxis will be missed by the other study designs.

### Exclusion criteria

• Reviews, discussion papers, non-research letters and editorials

• Qualitative studies

• Case studies

• Animal studies

### Study selection

The titles will be checked independently by two reviewers according to the above selection criteria and categorised as: included, not included and unsure. For those papers in the unsure category we will retrieve the abstract and re-categorise as above. Any discrepancies will be resolved by consensus and, if necessary, a third reviewer will be consulted. Full text copies of potentially relevant studies will be obtained and their eligibility for inclusion independently assessed. Studies that do not fulfil all of the inclusion criteria will be excluded.

### Quality assessment strategy

Quality assessments will independently be carried out on each study by two reviewers using the relevant version of the Critical Appraisal Skills Programme (CASP) quality assessment tool for systematic reviews [20]. We will assess the risk of bias of studies eligible for the review using the criteria suggested by EPOC [21]. RCTs, CCTs and CBAs will be assessed for generation of allocation sequence, concealment of allocation, baseline outcome measurements, baseline characteristics, incomplete outcome data, blinding of outcome assessor, protection against contamination, selective outcome reporting and other risks of bias. For ITS designs, we also assessed the independence of the intervention from other changes, the pre-specified shape of the intervention and if the intervention was unlikely to affect data collection. These will draw on the principles incorporated into the Cochrane EPOC guidelines for assessing intervention studies [22] and the Strengthening the Reporting of Observational Studies in Epidemiology for assessing observational studies [23]. Similarly, we will use the quality assessment form produced by the National Institute for Health and Clinical Excellence (NICE) to help critically appraise case series [24]. Any discrepancies will be resolved by discussion or, if agreement can not be reached, by arbitration by a third reviewer.

### Analysis, data synthesis and reporting

Data will be independently extracted onto a customised data extraction sheet by two reviewers, and any discrepancies will be resolved by discussion or, if agreement can not be reached, by arbitration by a third reviewer.

A descriptive summary with data tables will be produced to summarise the literature. If clinically and statistically appropriate, meta-analysis using either fixed-effect or random-effects modelling will be undertaken using methods suggested by Agresti and Coul [25]. A narrative synthesis of the data will also be undertaken.

This review has been registered with the International Prospective Register of Systematic Reviews (PROSPERO) and has registration number CRD42013003703 allocated to it. The Preferred Reporting Items for Systematic Reviews and Meta-Analyses (PRISMA) checklist will be used to guide the reporting of the systematic review [26].

## Discussion

The main strengths of this systematic review include the formal development of a review protocol, the range of databases searched and the formal critical appraisal of eligible studies. The preparatory work in developing the protocol involved extensive discussions with experts and based on these deliberations we decided a priori to consider lower forms of evidence in relation to epinephrine as expert opinion was in consensus that other more rigorous studies would be unethical/unfeasible to undertake. The main limitations are those inherent to systematic reviews, namely that it is not possible to guarantee that all relevant evidence was uncovered. This particularly needs to be borne in mind given the known limitations in relation to the sub-optimal coding of non-randomized study designs in biomedical databases. This risk was to an extent ameliorated by the extensive contact with experts with a view to identifying additional potentially relevant literature. We had hoped to be able to quantitatively synthesize data, but this was not possible because of the paucity of primary studies and the lack of comparable data. We therefore chose to present data descriptively and undertake a narrative summary of the evidence. There is at present little in the way of robust evidence to guide decisions on the acute and/or long-term management of anaphylaxis. Given the risk of death and the considerable morbidity associated with anaphylaxis these evidence gaps need to be filled wherever possible; this systematic review makes an attempt to do so.

## Abbreviations

CASP: Critical appraisal skills programme; CBA: Controlled before-and-after study; CCT: Controlled clinical trials; CDSR: Cochrane database of systematic reviews; DARE: Database of reviews of effectiveness; EAACI: European academy of allergy and clinical immunology; EED: Economic evaluations database; EPHPP: Effective public health practice project quality assessment tool; EPOC: Effective practice and organisation of care; HTA: Health technology assessments; ITS: Interrupted time-series study; NICE: National Institute for health and clinical excellence; OECD: Organization for economic co-operation and development; PROSPERO: Prospective register of systematic reviews; PRISMA: Preferred reporting items for systematic reviews and meta-analyses; RCT: Randomised controlled trial.

## Competing interests

The authors declare that they have no competing interests.

## Authors’ contributions

SD, SSP and TR conceptualised and designed the protocol and drafted earlier versions of the document in their capacity as methodologists. AM, GR and MW contributed to further refinements of the protocol and revised it critically for important intellectual content in their capacity as guideline leads. AS led on the development of concepts used in this protocol and revised it critically for important intellectual content in his capacity as the methodology lead. All authors approved the final version to be published.

## Supplementary Material

Additional file 1 Search strategies. Click here for file

## References

[B1] Johansson SGO Bieber T Dahl R Friedmann PS Lanier B Lockey RF Motala C Ortega Martell JA Platts-Mills TA Ring J Thien F Van Cauwenberge P Williams HC A revised nomenclature for allergy for global use: Report of the Nomenclature Review Committee of World Allergy Organization J Allergy Clin Immunol 2004 113 832 836 10.1016/j.jaci.2003.12.591 15131563

[B2] Muraro A Roberts G Clark A Eigenmann PA Halken S Lack G Moneret-Vautrin A Niggemann B Rancé F EAACI Task Force on Anaphylaxis in Children. The management of anaphylaxis in childhood: position paper of the European academy of allergology and clinical immunology Allergy 2007 62 8 857 871 10.1111/j.1398-9995.2007.01421.x 17590200

[B3] American Academy of Pediatrics Committee on School Health. Guidelines for urgent care in school Pediatrics 1990 86 999 1000 2251038

[B4] International Collaborative Study of Severe Anaphylaxis An epidemiologic study of severe anaphylactic and anaphylactoid reactions among hospital patients: methods and overall risks Epidemiology 1998 9 141 146 10.1097/00001648-199803000-00007 9504281

[B5] Australasian Society of Clinical Immunology and Allergy Inc. (ASCIA) Guidelines for EpiPen prescription.ASCIA Anaphylaxis Working Party 2004 2012 http://www.allergy.org.au/anaphylaxis/epipen_guidelines.htm

[B6] Joint Task Force on Practice Parameters; American Academy of Allergy, Asthma and Immunology; American College of Allergy, Asthma and Immunology; and Joint Council of Allergy, Asthma, and Immunology The diagnosis and management of anaphylaxis: an updated practice parameter J Allergy Clin Immunol 2005 115 3 suppl S483 S523 15753926 10.1016/j.jaci.2005.01.010

[B7] Sampson HA Muñoz-Furlong A Campbell RL Adkinson NF Bock A Branum A Brown SG Camargo CA Jr Cydulka R Galli SJ Gidudu J Gruchalla RS Harlor AD Jr Hepner DL Lewis LM Lieberman PL Metcalfe DD O’Connor R Muraro A Rudman A Schmitt C Scherrer D Simons FE Thomas S Wood JP Decker WW Second symposium on the definition and management of anaphylaxis: Summary report -Second National Institute of Allergy and Infectious Disease/Food Allergy and Anaphylaxis Network symposium J Allergy Clin Immunol 2006 117 391 397 10.1016/j.jaci.2005.12.1303 16461139

[B8] Walker S Sheikh A Managing anaphylaxis: effective emergency and long-term care are necessary Clin Exp Allergy 2003 33 8 1015 1018 10.1046/j.1365-2222.2003.01754.x 12911771

[B9] Pumphrey RS Lessons for management of anaphylaxis from a study of fatal reactions Clin Experimental Allergy 2000 30 8 1144 1150 10.1046/j.1365-2222.2000.00864.x 10931122

[B10] Bock S Munoz-Furlong A Sampson HA Fatalities due to anaphylactic reactions to foods J Allergy Clin Immunol 2001 107 1 10.1067/mai.2001.112458 11150011

[B11] Lieberman P Use of epinephrine in the treatment of anaphylaxis Curr Opin Allergy Clin Immunol 2003 3 4 313 318 10.1097/00130832-200308000-00013 12865777

[B12] Alrasbi M Sheikh A Comparison of international guidelines for the emergency medical management of anaphylaxis Allergy 2007 62 8 838 841 10.1111/j.1398-9995.2007.01434.x 17620061

[B13] Simons FE Sheikh A Evidence-based management of anaphylaxis Allergy 2007 62 8 827 829 10.1111/j.1398-9995.2007.01433.x 17620059

[B14] McMaster University Health Information Research Unit http://hiru.mcmaster.ca/hiru/HIRU_Hedges_MEDLINE_Strategies.aspx#Reviews

[B15] Higgins JPT, Green S Cochrane Handbook for Systematic Reviews of Interventions Version 5.1.0 (updated March 2011) 2012 Oxford, UK: The Cochrane Collaboration, 2011 http://www.cochrane-handbook.org

[B16] EPOC Grouphttp://epoc.cochrane.org/literature-searching-systematic-reviews

[B17] Cochrane Effective Practice & Organisation of Care (EPOC) Group Personal communication Michelle Fiander, Information Specialist & Trial Search Co-ordinator 2012 Ottawa, Canada: EPOC

[B18] Effective Practice and Organisation of Care Group What study designs should be included in an EPOC review and what should they be called 2012 http://epoc.cochrane.org/sites/epoc.cochrane.org/files/uploads/EPOC%20Study%20Designs%20About.pdf

[B19] OCEBM Levels of Evidence Working Group The Oxford 2011 Levels of Evidence.Oxford Centre for Evidence-Based Medicine 2012 Available online at http://www.cebm.net/index.aspx?o=5653 Last accessed on 28 September

[B20] CASP checklist for systematic reviews 2012 http://www.casp-uk.net/wp-content/uploads/2011/11/CASP_Systematic_Review_Appraisal_Checklist_14oct10.pdf

[B21] Effective Practice and Organisation of Care Group EPOC Website 2012 http://epoc.cochrane.org/cochrane-resources-0

[B22] Cochrane Effective Practice and Organisation of Care Group Methods papers 2012 http://epoc.cochrane.org/mecir-searching

[B23] Vandenbroucke JP Elm Eric V Altman DG Gotzsche PC Mulrow CD Pocock SJ Schlesselman JJ Egger M STROBE Initiative: Strengthening the Reporting of Observational Studies in Epidemiology (STROBE): explanation and elaboration PLoS Med 2007 4 1628 1654 10.1371/journal.pmed.0040297 PMC2020496 17941715

[B24] Higgins JPT Green S Cochrane Handbook for Systematic Reviews of Interventions. Version 5.0.2 (Chapter 11, Section 11) 2011 Oxford, UK: Cochrane Group

[B25] Agresti A Coull BA Approximate is better than “exact” for interval estimation of binomial proportions Am Statis 1998 52 119 126

[B26] Moher D Liberati A Tetzlaff J Altman DG The PRISMA Group Preferred Reporting Items for Systematic Reviews and Meta-Analyses: The PRISMA Statement PLoS Med 2009 6 6 e1000097 19621072 10.1371/journal.pmed.1000097 PMC2707599

