# Supplementary material for: The acute and long-term management of anaphylaxis: protocol for a systematic review
Source: Clin Transl Allergy. 2013 Apr 10;3:14. doi: 10.1186/2045-7022-3-14 (PMC3626654; doi:10.1186/2045-7022-3-14)
Supplement: Additional file 1 — Search strategies. [file 2045-7022-3-14-S1.docx]

**Additional file 1: Search strategies**

*Database: Ovid MEDLINE(R) In-Process & Other Non-Indexed Citations and Ovid MEDLINE(R) <1946 to Present>*

Search Strategy:

--------------------------------------------------------------------------------

| 1 | Anaphylaxis/ |
| --- | --- |
| 2 | anaphylaxis react*.mp. |
| 3 | anaphylactic react*.mp. |
| 4 | anaphylactic shock*.mp. |
| 5 | anaphylactoid syndrome*.mp. |
| 6 | anaphylactoid react*.mp. |
| 7 | anaphylactic syndrome*.mp. |
| 8 | anaphylactoid shock*.mp. |
| 9 | acute systemic allergic react*.mp. |
| 10 | idiopathic anaphylaxis.mp. |
| 11 | systemic anaphylaxis.mp. |
| 12 | or/1-11 |
| 13 | (rat or rats or cow or cows or chicken? or horse or horses or mice or mouse or bovine or animal?).ti. |
| 14 | exp animals/ not humans.sh. |
| 15 | 13 or 14 |
| 16 | 12 not 15 |
| 17 | MEDLINE.tw. |
| 18 | systematic review.tw. |
| 19 | meta analysis.pt. |
| 20 | or/17-19 |
| 21 | randomized controlled trial.pt. |
| 22 | controlled clinical trial.pt. |
| 23 | randomized.ab. |
| 24 | placebo.ab. |
| 25 | clinical trials as topic.sh. |
| 26 | randomly.ab. |
| 27 | trial.ti. |
| 28 | or/21-27 |
| 29 | intervention?.ti. or (intervention? adj6 (clinician? or collaborat$ or community or complex or DESIGN$ or doctor? or educational or family doctor? or family physician? or family practitioner? or financial or GP or general practice? or hospital? or impact? or improv$ or individuali?e? or individuali?ing or interdisciplin$ or multicomponent or multi-component or multidisciplin$ or multi-disciplin$ or multifacet$ or multi-facet$ or multimodal$ or multi-modal$ or personali?e? or personali?ing or pharmacies or pharmacist? or pharmacy or physician? or practitioner? or prescrib$ or prescription? or primary care or professional$ or provider? or regulatory or regulatory or tailor$ or target$ or team$ or usual care)).ab. |
| 30 | (pre-intervention? or preintervention? or "pre intervention?" or post-intervention? or postintervention? or "post intervention?").ti,ab. |
| 31 | (hospital$ or patient?).hw. and (study or studies or care or health$ or practitioner? or provider? or physician? or nurse? or nursing or doctor?).ti,hw. |
| 32 | demonstration project?.ti,ab. |
| 33 | (pre-post or "pre test$" or pretest$ or posttest$ or "post test$" or (pre adj5 post)).ti,ab. |
| 34 | (pre-workshop or post-workshop or (before adj3 workshop) or (after adj3 workshop)).ti,ab. |
| 35 | trial.ti. or ((study adj3 aim?) or "our study").ab. |
| 36 | (before adj10 (after or during)).ti,ab. |
| 37 | ("quasi-experiment$" or quasiexperiment$ or "quasi random$" or quasirandom$ or "quasi control$" or quasicontrol$ or ((quasi$ or experimental) adj3 (method$ or study or trial or design$))).ti,ab,hw. |
| 38 | (time series adj2 interrupt$).ti,ab,hw. |
| 39 | (time points adj3 (over or multiple or three or four or five or six or seven or eight or nine or ten or eleven or twelve or month$ or hour? or day? or "more than")).ab. |
| 40 | pilot.ti. |
| 41 | Pilot projects/ |
| 42 | (clinical trial or controlled clinical trial or multicenter study).pt. |
| 43 | (multicentre or multicenter or multi-centre or multi-center).ti. |
| 44 | random$.ti,ab. or controlled.ti. |
| 45 | (control adj3 (area or cohort? or compare? or condition or design or group? or intervention? or participant? or study)).ab. not (controlled clinical trial or randomized controlled trial).pt. |
| 46 | "commenton".cm. or review.ti,pt. or randomized controlled trial.pt. |
| 47 | (case$ and series).tw. |
| 48 | or/29-47 |
| 49 | 16 and 20 |
| 50 | 16 and 28 |
| 51 | 16 and 48 |
| 52 | 49 or 50 or 51 |

*Database: Embase Classic+Embase <1947 to 2012 September 18>*

Search Strategy:

--------------------------------------------------------------------------------

| 1 | Anaphylaxis/ |
| --- | --- |
| 2 | anaphylaxis react*.mp. |
| 3 | anaphylactic react*.mp. |
| 4 | anaphylactic shock*.mp. |
| 5 | anaphylactoid syndrome*.mp. |
| 6 | anaphylactoid react*.mp. |
| 7 | anaphylactic syndrome*.mp. |
| 8 | anaphylactoid shock*.mp. |
| 9 | acute systemic allergic react*.mp. |
| 10 | idiopathic anaphylaxis.mp. |
| 11 | systemic anaphylaxis.mp. |
| 12 | or/1-11 |
| 13 | (rat or rats or cow or cows or chicken? or horse or horses or mice or mouse or bovine or animal?).ti. |
| 14 | exp animals/ not humans.sh. |
| 15 | 13 or 14 |
| 16 | 12 not 15 |
| 17 | intervention?.ti. or (intervention? adj6 (clinician? or collaborat$ or community or complex or DESIGN$ or doctor? or educational or family doctor? or family physician? or family practitioner? or financial or GP or general practice? or hospital? or impact? or improv$ or individuali?e? or individuali?ing or interdisciplin$ or multicomponent or multi-component or multidisciplin$ or multi-disciplin$ or multifacet$ or multi-facet$ or multimodal$ or multi-modal$ or personali?e? or personali?ing or pharmacies or pharmacist? or pharmacy or physician? or practitioner? or prescrib$ or prescription? or primary care or professional$ or provider? or regulatory or regulatory or tailor$ or target$ or team$ or usual care)).ab. (172198) |
| 18 | (pre-intervention? or preintervention? or "pre intervention?" or post-intervention? or postintervention? or "post intervention?").ti,ab. [added 2.4] (10006) |
| 19 | (hospital$ or patient?).hw. and (study or studies or care or health$ or practitioner? or provider? or physician? or nurse? or nursing or doctor?).ti,hw. (1423864) |
| 20 | demonstration project?.ti,ab. |
| 21 | [or/17-42] |
| 22 | [or/44-46] |
| 23 | [or/48-62] |
| 24 | [or/67-69] |
| 25 | *anaphylactic shock/ |
| 26 | *anaphylaxis/ |
| 27 | anaphyla$.ti,ab. |
| 28 | or/25-27 |
| 29 | intervention?.ti. or (intervention? adj6 (clinician? or collaborat$ or community or complex or DESIGN$ or doctor? or educational or family doctor? or family physician? or family practitioner? or financial or GP or general practice? or hospital? or impact? or improv$ or individuali?e? or individuali?ing or interdisciplin$ or multicomponent or multi-component or multidisciplin$ or multi-disciplin$ or multifacet$ or multi-facet$ or multimodal$ or multi-modal$ or personali?e? or personali?ing or pharmacies or pharmacist? or pharmacy or physician? or practitioner? or prescrib$ or prescription? or primary care or professional$ or provider? or regulatory or regulatory or tailor$ or target$ or team$ or usual care)).ab. |
| 30 | (pre-intervention? or preintervention? or "pre intervention?" or post-intervention? or postintervention? or "post intervention?").ti,ab. [added 2.4] |
| 31 | (hospital$ or patient?).hw. and (study or studies or care or health$ or practitioner? or provider? or physician? or nurse? or nursing or doctor?).ti,hw. |
| 32 | demonstration project?.ti,ab. |
| 33 | (pre-post or "pre test$" or pretest$ or posttest$ or "post test$" or (pre adj5 post)).ti,ab. |
| 34 | (pre-workshop or post-workshop or (before adj3 workshop) or (after adj3 workshop)).ti,ab. |
| 35 | trial.ti. or ((study adj3 aim?) or "our study").ab. (711924) |
| 36 | (before adj10 (after or during)).ti,ab. |
| 37 | (time points adj3 (over or multiple or three or four or five or six or seven or eight or nine or ten or eleven or twelve or month$ or hour? or day? or "more than")).ab. |
| 38 | pilot.ti. |
| 39 | intervention?.ti. or (intervention? adj6 (clinician? or collaborat$ or community or complex or DESIGN$ or doctor? or educational or family doctor? or family physician? or family practitioner? or financial or GP or general practice? or hospital? or impact? or improv$ or individuali?e? or individuali?ing or interdisciplin$ or multicomponent or multi-component or multidisciplin$ or multi-disciplin$ or multifacet$ or multi-facet$ or multimodal$ or multi-modal$ or personali?e? or personali?ing or pharmacies or pharmacist? or pharmacy or physician? or practitioner? or prescrib$ or prescription? or primary care or professional$ or provider? or regulatory or regulatory or tailor$ or target$ or team$ or usual care)).ab. |
| 40 | (pre-intervention? or preintervention? or "pre intervention?" or post-intervention? or postintervention? or "post intervention?").ti,ab. [added 2.4] |
| 41 | (hospital$ or patient?).hw. and (study or studies or care or health$ or practitioner? or provider? or physician? or nurse? or nursing or doctor?).ti,hw. |
| 42 | demonstration project?.ti,ab. |
| 43 | (pre-post or "pre test$" or pretest$ or posttest$ or "post test$" or (pre adj5 post)).ti,ab. |
| 44 | (pre-workshop or post-workshop or (before adj3 workshop) or (after adj3 workshop)).ti,ab. |
| 45 | trial.ti. or ((study adj3 aim?) or "our study").ab. |
| 46 | (before adj10 (after or during)).ti,ab. |
| 47 | (time points adj3 (over or multiple or three or four or five or six or seven or eight or nine or ten or eleven or twelve or month$ or hour? or day? or "more than")).ab. |
| 48 | pilot.ti. |
| 49 | (multicentre or multicenter or multi-centre or multi-center).ti. (33919) |
| 50 | random$.ti,ab. or controlled.ti. |
| 51 | review.ti. [EM] |
| 52 | *experimental design/ or *pilot study/ or quasi experimental study/ [EM] |
| 53 | ("quasi-experiment$" or quasiexperiment$ or "quasi random$" or quasirandom$ or "quasi control$" or quasicontrol$ or ((quasi$ or experimental) adj3 (method$ or study or trial or design$))).ti,ab. [EM] |
| 54 | ("time series" adj2 interrupt$).ti,ab. [EM] |
| 55 | or/29-54 |
| 56 | meta-analys:.mp. |
| 57 | search:.tw. |
| 58 | review.pt. |
| 59 | or/56-58 |
| 60 | random$.tw. |
| 61 | factorial$.tw. |
| 62 | crossover$.tw. |
| 63 | cross over.tw. |
| 64 | cross-over.tw. |
| 65 | placebo$.tw. |
| 66 | (doubl$ adj blind$).tw. |
| 67 | singl$ adj blind$).tw. |
| 68 | assign$.tw. |
| 69 | allocat$.tw. |
| 70 | volunteer$.tw. |
| 71 | crossover procedure/ |
| 72 | double blind procedure/ |
| 73 | randomized controlled trial/ |
| 74 | single blind procedure/ |
| 75 | exp case study/ |
| 76 | (case$ and series).tw. |
| 77 | or/60-76 |
| 78 | 28 and 55 |
| 79 | 28 and 59 |
| 80 | 28 and 77 |
| 81 | or/78-80 |
| 82 | 81 not 15 |

*Database: CINAHL*

Search strategy:

| S1 or S2 or S3 | **Limiters** - Exclude MEDLINE records |
| --- | --- |
| S3 | "anaphylaxis" |
| S2 | "anaphylactic" |
| S1 | (MM "Anaphylaxis") |

*Database: ISI Web of Science: Science Citation Index, Conference Proceedings Citation*

Search strategy:

# 2

Refined by: Web of Science Categories=(ALLERGY OR IMMUNOLOGY) AND Document Types=( PROCEEDINGS PAPER OR MEETING ABSTRACT )

Databases=CPCI-S

Lemmatization=On

#1

Topic=((anaphylaxis or anaphylactic))

Databases=CPCI-S

Lemmatization=On

*Database: Cochrane Library*

Search strategy:

#1 MeSH descriptor Anaphylaxis explode all trees

#2 Anaphylactic

#3 Anaphylaxis

#4 (#1 OR #2 OR #3)

*Database: TRIP Database*

Search Stategy: (Advanced search screen)

area:"Allergies and Immunology"

any of these words: anaphylaxis or anaphylactic

Downloaded: Evidence Based Synopses, Systematic Reviews, Guidelines

*Database: Clinicaltrials.gov*

Search Strategy: (Advanced search screen)

Conditions: anaphylaxis or anaphylactic
